# Analyses of starch biosynthetic protein complexes and starch properties from developing mutant rice seeds with minimal starch synthase activities

**DOI:** 10.1186/s12870-018-1270-0

**Published:** 2018-04-10

**Authors:** Mari Hayashi, Naoko Crofts, Naoko F. Oitome, Naoko Fujita

**Affiliations:** Department of Biological Production, Akita Prefectural University, 241-438 Kaidobata-Nishi, Shimoshinjo Nakano, Akita City, Akita 010–0195 Japan

**Keywords:** Amylopectin, Amylose, Starch synthase, Protein complexes, Rice (*Oryza sativa*)

## Abstract

**Background:**

Starch is the major component of cereal grains and is composed of essentially linear amylose and highly branched amylopectin. The properties and composition of starch determine the use and value of grains and their products. Starch synthase (SS) I, SSIIa, and SSIIIa play central roles in amylopectin biosynthesis. These three SS isozymes also affect seed development, as complete loss of both SSI and SSIIIa under reduced SSIIa activity in rice lead to sterility, whereas presence of minimal SSI or SSIIIa activity is sufficient for generating fertile seeds. SSs, branching enzymes, and/or debranching enzymes form protein complexes in cereal. However, the relationship between starch properties and the formation of protein complexes remain largely unknown. To better understand this phenomenon, properties of starch and protein complex formation were analyzed using developing mutant rice seeds (*ss1*^*L*^*/ss2a*^*L*^*/ss3a*) in which all three major SS activities were reduced.

**Results:**

The SS activity of *ss1*^*L*^*/ss2a*^*L*^*/ss3a* was 25%–30% that of the wild-type. However, the grain weight of *ss1*^*L*^*/ss2a*^*L*^*/ss3a* was 89% of the wild-type, 55% of which was starch, showing considerable starch synthesis. The reduction of soluble SS activity in *ss1*^*L*^*/ss2a*^*L*^*/ss3a* resulted in increased levels of ADP-glucose pyrophosphorylase and granule-bound starch synthase I, which are responsible for substrate synthesis and amylose synthesis, respectively. Together, these features led to an increase in apparent amylose content (34%) in *ss1*^*L*^*/ss2a*^*L*^*/ss3a* compared with wild-type (20%). Gel filtration chromatography of the soluble proteins in *ss1*^*L*^*/ss2a*^*L*^*/ss3a* showed that the majority of the starch biosynthetic enzymes maintained the similar elution patterns as wild-type, except that the amounts of high-molecular-weight SSI (> 300 kDa) were reduced and SSIIa of approximately 200–300 kDa were present instead of those > 440 kDa, which predominate in wild-type. Immuno-precipitation analyses suggested that the interaction between the starch biosynthetic enzymes maybe reduced or weaker than in wild-type.

**Conclusions:**

Although major SS isozymes were simultaneously reduced in *ss1*^*L*^*/ss2a*^*L*^*/ss3a* rice, active protein complexes were formed with a slightly altered pattern, suggesting that the assembly of protein complexes may be complemented among the SS isozymes. In addition, *ss1*^*L*^*/ss2a*^*L*^*/ss3a* maintained the ability to synthesize starch and accumulated less amylopectin and more amylose in starch.

**Electronic supplementary material:**

The online version of this article (10.1186/s12870-018-1270-0) contains supplementary material, which is available to authorized users.

## Background

Starch is a high-molecular-weight glucose polymer consisting of essentially linear amylose and highly branched amylopectin. Starch utilized for food and industrial applications is primarily accumulated in the storage tissues of green plants. In cereals, starch accumulation is directly associated with yield and is the most important agricultural trait. Starch is synthesized through the concerted activities of at least four classes of enzymes [1–5]: starch synthase (SS; *EC* 2.4.1.21), which functions in the elongation of α-1,4 linear chains of starch using adenosine diphosphate (ADP)-glucose as a substrate produced by ADP-glucose pyrophosphorylase (AGPase; *EC* 2.7.7.27); starch branching enzyme (BE; *EC* 2.4.1.18), which forms α-1,6 branch points of amylopectin; debranching enzymes (DBE), which removes improper branches in amylopectin such as isoamylase (ISA; *EC* 3.2.1.68) and pullulanase (PUL; *EC* 3.2.1.41). In addition to these enzymes, phosphorylase (Pho; *EC* 2.4.1.1) is thought to be involved in the initiation process of starch biosynthesis [6–8]. Multiple isozymes are present in these four classes of enzymes in higher plants.

The SS family has the largest number of isozymes encoded by eleven different genes in most rice cultivars. Among these, SSI, SSIIa, SSIIIa, and GBSSI are highly expressed in the developing rice endosperm [9, 10]. Rice cultivars are divided into two groups; indica and japonica cultivars. The activity of SSIIa in the japonica cultivars is only 10% of that of the indica cultivar due to the three single nucleotide polymorphisms (SNPs) in the gene [11]. Since SSIIa is almost inactive in japonica cultivars (*SS1/ss2a*^*L*^*/SS3a*) [11], SSI and SSIIIa account for 60% and 30% of the SS activity, respectively, in the soluble fraction of the developing endosperm. Thus, SSI and SSIIIa are mainly responsible for elongation of amylopectin chains in japonica cultivars [12]. We have isolated multiple allelic *ss1* mutant lines from a retrotransposon (*Tos17*) insertion line derived from the japonica rice cultivar, Nipponbare (*SS1/ss2a*^*L*^*/SS3a*). Among these, the *ss1* null mutant (*e7*: *ss1/ss2a*^*L*^*/SS3a*) has no SSI activity due to the insertion of *Tos17* into exon 7 of the *SSI* gene, and the *ss1* leaky mutant (*ss1*^*L*^) (*i2–1*: *ss1*^*L*^*/ss2a*^*L*^*/SS3a*) has only 20% of the SSI activity of the wild-type (*SS1/ss2a*^*L*^*/SS3a*) due to the insertion of *Tos17* into intron 2 of the *SSI* gene [12]. We have also isolated the *ss3a* null mutant line (*e1*: *SS1/ss2a*^*L*^*/ss3a*), which has no SSIIIa activity due to the insertion of *Tos17* into exon 1 of the *SSIIIa* gene [13]. A complete deficiency of both SSI and SSIIIa activities in the rice *ss1/ss3a* null double mutant (*e7* x *e1*: *ss1/ss2a*^*L*^*/ss3a*) led to sterility. However, opaque seeds were obtained in mutants heterozygous for one of these genes (*ss1ss1/ ss2a*^*L*^*ss2a*^*L*^*/SS3ass3a* or *SS1ss1/ ss2a*^*L*^*ss2a*^*L*^*/ss3ass3a*) [14]. These findings suggest that either SSI or SSIII is essential for starch biosynthesis in conditions of low SSIIa activity such as occurs in japonica rice endosperm. Moreover, the double recessive mutant line produced by crossing the leaky *ss1* mutant [12] with the *ss3a* null mutant (*i2–1* x *e1*) [13] was fertile [14]. The endosperm starch granules of this mutant were round in comparison to the parent mutant lines and wild-type, with several gaps between the starch granules and amyloplasts [15]. The chain-length profile of *ss1*^*L*^*/ss2a*^*L*^*/ss3a* endosperm starch by capillary electrophoresis was essentially similar to that of *SS1/ss2a*^*L*^*/ss3a* [14, 15]; the levels of amylopectin branch chains with DP ≤10 and 30 ≤ DP ≤60 were reduced, and the levels of chains with 11 ≤ DP ≤15 were higher, compared with wild-type (*SS1/ss2a*^*L*^*/SS3a*). By contrast, the levels of chains with 16 ≤ DP ≤24 were also higher in *ss1*^*L*^*/ss2a*^*L*^*/ss3a* than in wild-type (*SS1/ss2a*^*L*^*/SS3a*), indicating that the chain-length distribution pattern of the double mutant is an additive trait from both parent mutant lines [14]. However, the alteration in the chain-length profile of *ss1*^*L*^*/ss2a*^*L*^*/ss3a* was more pronounced in the range of DP ≤20 than that calculated by adding the profiles of the *ss1*^*L*^*/ss2a*^*L*^*/SS3a* and *SS1/ss2a*^*L*^*/ss3a* lines, indicating that the reduced SSI activity has an additive effect on the chain-length distribution of amylopectin in the *SS1/ss2a*^*L*^*/ss3a* background, with a slight synergistic enhancement [14, 15]. The gelatinization temperature of *ss1*^*L*^*/ss2a*^*L*^*/ss3a* starch was 3 °C higher than that of wild-type (*SS1/ss2a*^*L*^*/SS3a*), and the viscosity of gelatinized starch was lower than that of wild-type (*SS1/ss2a*^*L*^*/SS3a*) [15].

Starch biosynthetic enzymes in developing cereal endosperms have been shown to associate as protein-protein complexes [16–23], and some of those interactions are regulated in a phosphorylation-dependent manner [24]. SS isozymes, including SSI, SSIIa and SSIIIa, in developing rice endosperm also form high-molecular-weight protein complexes with starch biosynthesis activities [23]. The analyses of protein complex formation from reduced SS activities should provide valuable information for understanding the relationships between protein complex formation and starch biosynthesis.

In the present study, we analyzed the detailed structure of endosperm starch by gel-filtration chromatography in the *ss1*^*L*^*/ss2a*^*L*^*/ss3a*, as well as the effects of low SS activity in the soluble fraction on the protein complex component during starch biosynthesis and the activities of other starch biosynthetic enzymes.

## Methods

### Plant materials

The *ss1*^*L*^*/ss2a*^*L*^*/ss3a* [14] was generated by crossing a leaky mutant for SSI activity (*ss1*^*L*^*/ss2a*^*L*^*/SS3a*, *i2–1*) [12] with a null mutant for SSIIIa activity (*SS1/ss2a*^*L*^*/ss3a*, *e1*) [13]. The parental cultivar Nipponbare (*SS1/ss2a*^*L*^*/SS3a*, Nip) and parental mutant lines (*ss1*^*L*^*/ss2a*^*L*^*/SS3a* and *SS1/ss2a*^*L*^*/ss3a*) were also used in the experiments. Rice plants were grown during the summer of 2009 (for gel filtration chromatography of starch) and 2013 (for all experiments) in an experimental paddy field at Akita Prefectural University, Japan, under natural environmental conditions.

### Starch granule purification

Starch granules were purified from polished mature rice seeds free of embryos using the cold-alkali method [25, 26].

### Starch contents in grains

The starch contents of rice seeds were measured using the methods of Fujita et al. [27].

### Native-PAGE/activity staining

Native-PAGE/activity staining of SS and DBE were performed as described by Abe et al. [28]. BE activity staining was performed on 5% (*w*/*v*) acrylamide slab gels containing 0.0001% (w/v) oyster glycogen (G8751, Sigma) according to Abe et al. [28], with the addition of glucose-1-phosphate at a final concentration of 0.1 M. AGPase activity was quantified as described previously [29].

### Enzyme assays

Total SS activities in the soluble proteins were measured as described by Nishi et al. [30] with some modifications. The initial SS reaction was performed in a total volume of 80 μL consisting 50 mM bicine-NaOH, pH 7.4, 0 or 250 mM citrate-NaOH, pH 7.5, 20 mM DTT, 8 mM ADP-glucose, 0 or 2 mg/mL oyster glycogen, and 8 μL of soluble extract prepared by extracting approximately 12 mg of developing rice endosperm with 100 μL of buffer containing 50 mM imidazole-HCl, pH 7.4, 8 mM MgCl_2_, 5 mM DTT, and 10 μL/mL protease inhibitor cocktail (Sigma). Reactions were performed at 30 °C and terminated at 0 or 20 min by boiling. Secondary reactions were performed in a total volume of 400 μL containing 12.5 mM HEPES-NaOH, pH 7.4, 2.5 mM phosphocreatine, 50 mM KCl, 25 mM MgCl_2_, 5 mM DTT, 0.3 units of creatine kinase, and the SS reaction. The final citrate concentration was equalized since the presence of citrate interferes with the secondary reaction. The mixture was incubated at 37 °C overnight, terminated by boiling and centrifuged. The final reactions were performed in 500 μL containing 300 μL of the second reaction and 50 mM HEPES-NaOH, pH 7.4, 8 mM MgCl_2_, 4 mM glucose, and 0.4 mM NADP^+^. The maximum O.D. at 340 nm was measured after addition of hexokinase (0.5 U) and glucose-6-phosphate dehydrogenase (1.5 μg).

### Immuno-blotting

Immuno-blotting was performed as described by Crofts et al. [23, 31] using antiserum raised against SSI, SSIIa, SSIIIa, SSIVb, GBSSI, BEI, BEIIa, BEIIb, ISA1, PUL, and Pho1.

### Gel filtration chromatography of proteins and immuno-precipitation

Gel filtration chromatography was performed using mid-developing seeds (DAF12–15) as described [23]. The eluted materials were analyzed by zymogram and immuno-blotting after Native-PAGE and SDS-PAGE as described [23]. Immuno-precipitation was also performed as described [23]. The control experiments were performed using pre-immune serum.

### Size fractionation of starch and amylose content analysis

Gel filtration chromatography of debranched starch was performed as described [31].

## Results

### Estimation of the remaining soluble SS activity in the double mutant by Native-PAGE

**Fig. 1:**
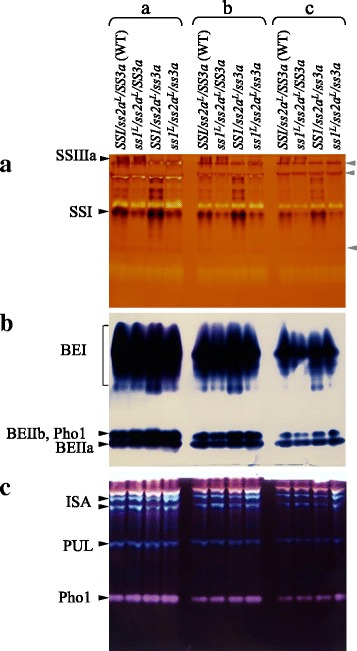
Zymograms of soluble starch biosynthetic enzyme activities. Native-PAGE/activity staining of starch synthases (SSs; Panel **a**), branching enzymes (BEs; Panel **b**), and debranching enzymes (DBEs; Panel **c**) using developing endosperm of double mutant, parental mutant, and wild-type lines. Black arrowheads and brackets indicate SSI, SSIIIa, BEI, BEIIa, BEIIb, ISA (isoamylase), PUL (pullulanase), and Pho1 (phosphorylase) activity bands. Gray arrowheads in A indicate glycosyl hydrolase or transferase activities. Soluble protein extracts were prepared from developing endosperm at 12 days after flowering. The volumes of crude extract applied to the native gels in section ‘a’ were 2-fold and 4-fold greater than those applied in sections ‘b’ and ‘c’, respectively

To estimate the remaining soluble SS activity in the double mutant, two distinct methods were utilized. First, semi-quantitative native-polyacrylamide gel electrophoresis (PAGE) was performed using the soluble protein fraction from developing endosperm at 12 days after flowering (DAF) (Fig. 1a). This method visualizes the activities of specific SS isozymes, particularly SSI and SSIIIa. Lanes marked “a” contain twice the amount of samples in lanes marked “b” and four times the amount in lanes marked “c”. The intensity of the SSI activity band of *ss1*^*L*^*/ss2a*^*L*^*/SS3a* in “a” was similar to that of wild-type in “b”, although “b” contained only half of the volume of soluble protein loaded onto lane “a”, indicating that the SSI activity of *ss1*^*L*^*/ss2a*^*L*^*/SS3a* was ca. 50% of wild-type. The SSIIIa activity band was completely absent in *ss1*^*L*^*/ss2a*^*L*^*/ss3a*. SSI and SSIIIa activities in developing endosperm of wild-type japonica rice (*SS1/ss2a*^*L*^*/SS3a*) analyzed by zymogram are estimated to be 50%–60% and 30% of the total SS activities, respectively [13, 14]. Therefore, the remaining SS activity in *ss1*^*L*^*/ss2a*^*L*^*/ss3a* was estimated to be approximately 25%–30% of the total SS activity of the wild-type (*SS1/ss2a*^*L*^*/SS3a*).

### Estimating the remaining soluble SS activity in the double mutant by measuring total SS activity

The bulk of the SS activity in the soluble extract from developing endosperm was measured in the test tubes in the presence or absence of citrate and/or glycogen (Table 1) because citrate stimulates SSI and SSIIa, but not SSIIIa [32–34], and glucan primers are necessary for SSIIIa, but not for SSI [12, 34]. The requirements of citrate and glucan primers for other SS isozymes are currently unknown. The results showed that total SS activities were higher for all lines in the presence of both citrate and glycogen in comparison to the absence of either citrate or glycogen.

**Table 1:** Starch synthase and AGPase activity in developing seeds (10–15 days after flowering) of double mutants, their parental lines, and wild-type

	Starch synthase			AGPase
Line	μmol min^−1^ endosperm^-1 a^			μmol min^− 1^ endosperm^-1, a^
	+ Citrate +Glycogen	+ Citrate -Glycogen	- Citrate + Glycogen	
*SS1/ss2a*^*L*^*/SS3a* (Wild-type; Nipponbare)	11.09 ± 1.2 (100) ^b^	2.22 ± 0 (100) ^c^	1.17 ± 0 (100) ^d^	0.259 ± 0.020 ^**^ (100) ^e^
*ss1* ^*L*^ */ss2a* ^*L*^ */SS3a* (*i2–1*)	5.28 ± 0.1 (48)	N.D. (0)	1.04 ± 0.2 (89)	0.245 ± 0.014^**^ (95)
*SS1/ss2a* ^*L*^ */ss3a* (*e1*)	13.23 ± 0.5 (119)	5.36 ± 0.1 (241)	1.62 ± 0.1 (139)	0.324 ± 0.006^*, **^ (125)
*ss1* ^*L*^ */ss2a* ^*L*^ */ss3a* (*#6002*)	6.67 ± 0.6 (60)	N.D. (0)	0.76 ± 0.4 (73)	0.506 ± 0.031^*^ (196)

*ND*, Not detected

^a^Mean ± SE. *n* = 3 for all lines

^b, c, d, e^Percentage of wild-type, Nipponbare

^*^Significant differences between Nipponbare and mutant lines by the *t*-test at *P* < 0.05

^**^Significant differences between double mutant line and the other lines by the *t*-test at *P* < 0.05

In the presence of both citrate and glycogen, the total SS activity of *ss1*^*L*^*/ss2a*^*L*^*/SS3a* was 48% of wild-type, while that of *SS1/ss2a*^*L*^*/ss3a* and *ss1*^*L*^*/ss2a*^*L*^*/ss3a* was 119% and 60% of wild-type, respectively (Table 1). SS activity of *ss1*^*L*^*/ss2a*^*L*^*/ss3a* was 1.2-fold higher than that of *ss1*^*L*^*/ss2a*^*L*^*/SS3a*.

In the presence of citrate and the absence of glycogen—in which most of the SS activity is derived from SSI—, SS activities of *ss1*^*L*^*/ss2a*^*L*^*/SS3a* and *ss1*^*L*^*/ss2a*^*L*^*/ss3a* were below the detection limit, perhaps due to low abundance of SSI, while that of *SS1/ss2a*^*L*^*/ss3a* was 241% of wild-type (Table 1). These findings were consistent with an earlier observation [12]. In the absence of citrate and the presence of glucan primer—in which most of the SS activities derived from SSIIIa but also minor SSI activity, is detected —, SS activities of *SS1/ss2a*^*L*^*/ss3a* were 139% of wild-type, while that of *ss1*^*L*^*/ss2a*^*L*^*/ss3a* (73% of the wild-type) was lower than *ss1*^*L*^*/ss2a*^*L*^*/SS3a* (89% of wild-type) (Table 1).

### The effects of reduced SS activity on other starch biosynthetic enzymes

Next, the effects of the reduced SS activity in the soluble protein fraction on the activities of other starch biosynthetic enzymes were analyzed by semi-quantitative native-PAGE/activity staining (Fig. 1b; c). The activities of BEs (BEI, BEIIa and BEIIb), DBEs (ISA1 and PUL), and Pho1 in *ss1*^*L*^*/ss2a*^*L*^*/ss3a* were not significantly different from those of wild-type. By contrast, the AGPase activity of *SS1/ss2a*^*L*^*/ss3a* and *ss1*^*L*^*/ss2a*^*L*^*/ss3a* was 1.25- and 1.96-times higher than that of wild-type, respectively (Table 1).

### Expression levels of starch biosynthetic enzymes in developing rice seeds and the degree of binding to starch granules

**Fig. 2:**
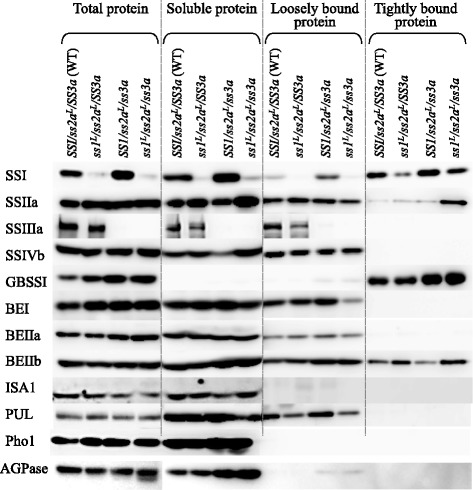
Enzyme distributions in protein fractions from developing endosperm. Immuno-blotting of total protein extract, the soluble protein fraction, the loosely bound protein fraction, and the tightly bound protein fraction from developing endosperm at 12 days after flowering using the indicated antibodies

To further examine the expression of starch biosynthetic isozymes in the rice endosperm and the degree of starch granule binding, total protein, soluble protein, loosely bound protein, and tightly bound protein were extracted from the developing rice seeds (see Methods) and analyzed by immuno-blotting using antiserum against each starch biosynthetic enzyme (Fig. 2). The results of immuno-blotting of total and soluble fractions using SSI antibodies were correlated to the strength of SSI activity from the soluble protein fractions (Fig. 1a); that is, the strongest and weakest SSI bands were detected in *SS1/ss2a*^*L*^*/ss3a* and *ss1*^*L*^*/ss2a*^*L*^*/SS3a*, respectively, among the four lines examined, and the bands from *ss1*^*L*^*/ss2a*^*L*^*/ss3a* were slightly more intense than those from *ss1*^*L*^*/ss2a*^*L*^*/SS3a* (Fig. 2). SSIIa bands from the total protein fractions of the three mutant lines were more intense than those of wild-type. Among the soluble protein fractions, the SSIIa band from *ss1*^*L*^*/ss2a*^*L*^*/ss3a* was the most intense, followed by *ss1*^*L*^*/ss2a*^*L*^*/SS3a*. Among the tightly bound protein fractions, the SSIIa band from *ss1*^*L*^*/ss2a*^*L*^*/ss3a* was extremely intense, whereas SSIIa levels in the loosely bound protein fractions were similar in all four lines. These results implied that some SSIIa protein in the double mutant was tightly bound to starch granules. SSIIIa was absent from *SS1/ss2a*^*L*^*/ss3a* and *ss1*^*L*^*/ss2a*^*L*^*/ss3a*, and was not detected in the tightly bound protein fractions. Most of the GBSSI protein was detected in the tightly bound protein fractions, and the GBSSI bands were stronger in *SS1/ss2a*^*L*^*/ss3a* and *ss1*^*L*^*/ss2a*^*L*^*/ss3a* than in wild-type and *ss1*^*L*^*/ss2a*^*L*^*/SS3a*. BEI bands were detected in the soluble protein and loosely bound protein fractions. The BEI band in the loosely bound protein fraction was weaker in *ss1*^*L*^*/ss2a*^*L*^*/ss3a* than in the other lines. BEIIb bands were detected in every fraction. Among the tightly bound protein fractions, the BEIIb band in *ss1*^*L*^*/ss2a*^*L*^*/SS3a* and *ss1*^*L*^*/ss2a*^*L*^*/ss3a* was more intense than those of wild-type and *SS1/ss2a*^*L*^*/ss3a*. By contrast, among loosely bound protein fractions, the PUL band from *ss3a* was more intense than those of the other lines. There was no significant difference in the levels of the other isozymes (SSIVb, BEIIa, ISA1, and Pho1) among the four lines in all fractions examined (Fig. 2).

### Molecular weight distributions of starch biosynthetic protein complexes in rice lines with reduced SS activity

**Fig. 3:**
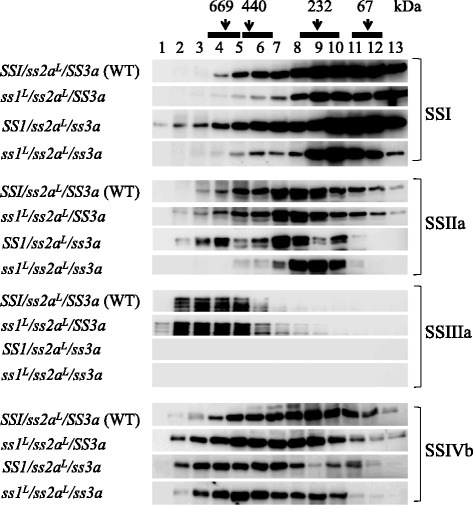
Molecular weight distributions of SS isozymes from developing rice endosperm determined by gel filtration chromatography. Soluble proteins were separated by gel filtration chromatography. Fractions were denatured and separated by SDS-PAGE, and immuno-blotting was performed using the indicated antibodies

**Fig. 4:**
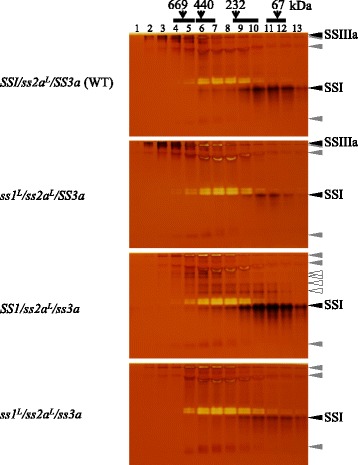
SS activities were visualized by non-denaturing zymograms using rice starch biosynthetic enzymes separated by gel filtration chromatography. Black arrowheads show the activities of the indicated SS isozymes. Gray arrowheads represent glycosyl hydrolase or glucan transferase activities. White arrowheads are SS activity bands found only in *ss3a*

To determine the effects of the SSIIIa deficiency and reduced SSI and SSIIa activities on the protein-protein complex formation of starch biosynthetic enzymes, crude extract from developing endosperm from *ss1*^*L*^*/ss2a*^*L*^*/ss3a* and the parental lines was subjected to gel filtration chromatography. The fractions were analyzed by denaturing SDS-PAGE, native-PAGE activity staining and their respective immuno-blotting (Figs. 3 and 4, Additional file 1).

To determine the size distributions of specific starch biosynthetic enzymes present in the protein complexes of developing rice endosperm, the eluted materials from gel filtration chromatography were denatured, separated by SDS-PAGE and detected using isozyme specific antibodies (Fig. 3 and Additional file 1). The strength of the immuno-blot signals on the different membranes was equalized using the loading control to compare the signals from the different lines.

The elution patterns and amounts of SSI were considerably different among the analyzed lines (Fig. 3). *ss1*^*L*^*/ss2a*^*L*^*/SS3a* and *ss1*^*L*^*/ss2a*^*L*^*/ss3a* had lower SSI signals in all fractions compared with wild-type, particularly in Fr. 4 to 8 (< 700 kDa), while *SS1/ss2a*^*L*^*/ss3a* showed stronger SSI signals, particularly in Fr. 2–5 (> 700 kDa) (Fig. 3). The elution patterns of SSIIa in *ss1*^*L*^*/ss2a*^*L*^*/SS3a* were similar to those of wild-type, while those of *SS1/ss2a*^*L*^*/ss3a* and *ss1*^*L*^*/ss2a*^*L*^*/ss3a* were different (Fig. 3). The SSIIa signal of *SS1/ss2a*^*L*^*/ss3a* in Fr. 4 was increased, but those of Fr. 5 and 6 were decreased (Fig. 3). The amount of SSIIa in the soluble fraction was increased in *ss1*^*L*^*/ss2a*^*L*^*/ss3a* compared with wild-type (Fig. 2, Soluble protein). However, the majority of SSIIa was eluted in Fr. 8 to 10 (Fig. 3). SSIIIa was eluted in Fr. 2 to 6 (> 440 kDa) in wild-type and *ss1*^*L*^*/ss2a*^*L*^*/SS3a*, but it was absent in *SS1/ss2a*^*L*^*/ss3a* and *ss1*^*L*^*/ss2a*^*L*^*/ss3a* as expected (Fig. 3). SSIIIa signals were detected as multiple bands, but all of these bands were absent in *SS1/ss2a*^*L*^*/ss3a* and *ss1*^*L*^*/ss2a*^*L*^*/ss3a*, confirming that the bands were derived from SSIIIa (Fig. 3). The elution patterns of SSIVb in *ss1*^*L*^*/ss2a*^*L*^*/SS3a*, *SS1/ss2a*^*L*^*/ss3a* and *ss1*^*L*^*/ss2a*^*L*^*/ss3a* were shifted towards a high molecular weight. The amount of SSIVb in Fr. 3 and 4 (> 700 kDa) in *SS1/ss2a*^*L*^*/ss3a* and in Fr. 4 to 6 in *ss1*^*L*^*/ss2a*^*L*^*/ss3a* were increased (Fig. 3)*.* The elution patterns of BEs and DBEs were similar among wild-type, *ss1*^*L*^*/ss2a*^*L*^*/SS3a* and *SS1/ss2a*^*L*^*/ss3a*, but the amounts of these enzymes in *ss1*^*L*^*/ss2a*^*L*^*/ss3a* were slightly reduced (Additional file 1). Overall, a reduction of major SS activities, namely, SSI and SSIIIa, under a low SSIIa background altered complex formation of SS isozymes such as SSI, SSIIa, and SSIVb.

### Native-PAGE/activity staining and immuno-blotting of protein complexes separated by gel filtration chromatography

To assess whether starch biosynthetic enzymes eluted in the high-molecular-weight fractions retained their activities, the fractions eluted from the gel filtration column were concentrated and analyzed by Native-PAGE activity staining (Fig. 4).

SSI activity was observed in Fr. 6 to 13 (43 to 440 kDa) in wild-type and *SS1/ss2a*^*L*^*/ss3a*, and the activity of SSI in those fractions was higher in *SS1/ss2a*^*L*^*/ss3a* compared with wild-type (Fig. 4). SSI activity in *ss1*^*L*^*/ss2a*^*L*^*/SS3a* and *ss1*^*L*^*/ss2a*^*L*^*/ss3a* was barely detectable in Fr. 9 to 13 (43–153 kDa) (Fig. 4). SSI activity was correlated to the amounts of SSI in each fraction (Figs. 3 and 4) and the expression levels of SSI in each line (Fig. 2). SSIIIa activity was observed in Fr. 2 to 5 (> 440 kDa) in wild-type and *ss1*^*L*^*/ss2a*^*L*^*/SS3a*, and SSIIIa activity of *ss1*^*L*^*/ss2a*^*L*^*/SS3a* in Fr. 3 and 4 was stronger than that of wild-type (Fig. 4). SSIIIa activity was absent in *SS1/ss2a*^*L*^*/ss3a* and *ss1*^*L*^*/ss2a*^*L*^*/ss3a* as expected (Fig. 4). Brown activity bands indicated in gray were also detected in the absence of ADP-glucose (Additional file 2), suggesting that they were the outcome of the activities of glycosyl hydrolase or glucan transferase, not starch synthase [23]. *SS1/ss2a*^*L*^*/ss3a* had additional SS activity bands indicated with white arrowheads, and these were likely to be protein complexes containing SSI as confirmed by immunoblotting (Fig. 4, Additional file 3). Immuno-blotting of the corresponding SS Native-PAGE gel confirmed that the indicated activity bands were SSI and SSIIIa (Additional file 3). Multiple SSI immuno-blot signals were detected in Fr. 5–9 (153–440 kDa) of *SS1/ss2a*^*L*^*/ss3a* (white arrowheads in Additional file 3). Although SSIIa and SSIVb activity could not be detected by zymogram (Fig. 4), both proteins were present on the native-PAGE gel (Additional file 3). SSIVb migrated closely to the very fast migrating band indicated in gray (Additional file 3). The activities of BEs, DBEs and Pho1 on the native-PAGE gel and their corresponding immuno-blotting of all lines were essentially the same as wild-type [23] (data not shown). Differences in western blotting patterns after native-PAGE and SDS-PAGE may be a result of less abundant differentially migrating proteins on native-PAGE that were below the detection limit of western blotting.

### Co-immuno-precipitation analyses to determine the association of starch biosynthetic enzymes in developing rice endosperm

**Fig. 5:**
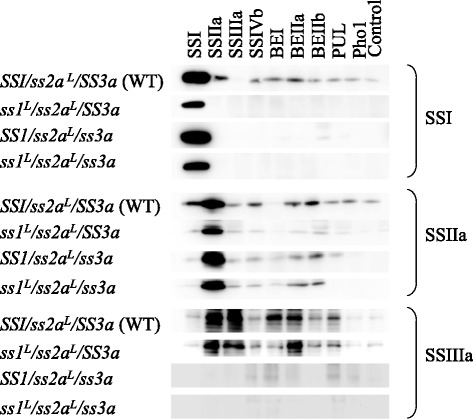
Analyses of protein-protein interactions between rice starch biosynthetic isozymes by co-immunoprecipitation. Immuno-precipitation experiments were performed using the isozyme specific antibodies indicated above and the soluble protein extract from wild-type, *ss1*^*L*^*/ss2a*^*L*^*/SS3a*, *SS1/ss2a*^*L*^*/ss3a* and *ss1*^*L*^*/ss2a*^*L*^*/ss3a*. Immuno-blotting was performed using the antibodies indicated on the right

To assess the differences in compositions of starch biosynthetic protein complexes, co-immuno-precipitation experiments were performed using soluble proteins obtained from developing rice seeds of wild-type, *ss1*^*L*^*/ss2a*^*L*^*/SS3a*, *SS1/ss2a*^*L*^*/ss3a*, *ss1*^*L*^*/ss2a*^*L*^*/ss3a*, and isozyme specific antibodies. The control experiments were performed using pre-immune serum. The results obtained for wild-type were generally consistent with a previous study showing the interaction of SSI-SSIIa, SSIIa-SSIIIa, and BEIIb-SSIIa, (Fig. 5 and [23]); however, the associations of SS isozymes with other isozymes were not detected in *ss1*^*L*^*/ss2a*^*L*^*/SS3a*, *SS1/ss2a*^*L*^*/ss3a* and *ss1*^*L*^*/ss2a*^*L*^*/ss3a* (Fig. 5). The interactions between BEI-BEIIb, BEI-PUL and Pho1-BEIIa in all lines were essentially the same as in wild-type [23] (Additional file 4). The immuno-precipitation results suggested alterations in protein complex formation, with potentially weaker interactions in the mutant lines.

### The effects of reduced SS activity on seed morphology, grain weight, and starch contents

**Fig. 6:**
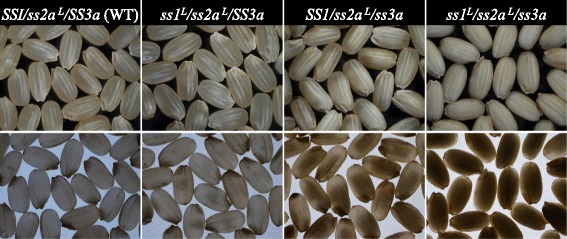
Seed morphologies of mutant and wild-type lines. The morphology of mature rice seeds was observed using a stereomicroscope with an overhead light (upper panels) and on a light box (lower panels)

Wild-type and *ss1*^*L*^*/ss2a*^*L*^*/SS3a* and *SS1/ss2a*^*L*^*/ss3a* seeds exhibited a translucent and white core morphology, respectively [12, 13]. By contrast, *ss1*^*L*^*/ss2a*^*L*^*/ss3a* seeds were opaque, as were the seeds of lines that were heterozygous for one of these genes (*ss1ss1/SS3ass3a* or *SS1ss1/ss3ass3a*) (Fig. 6 and [13]).

The grain weight of *ss1*^*L*^*/ss2a*^*L*^*/SS3a* and *SS1/ss2a*^*L*^*/ss3a* was 100% and 93%, respectively, of wild-type (Table 2). However, while the grain weight of *ss1*^*L*^*/ss2a*^*L*^*/ss3a* was 89% of wild-type, the SS activity in the soluble fraction of this mutant was only 20% of wild-type (Fig. 1). The starch contents of *ss1*^*L*^*/ss2a*^*L*^*/SS3a*, *SS1/ss2a*^*L*^*/ss3a*, and *ss1*^*L*^*/ss2a*^*L*^*/ss3a* were 58%, 54%, and 55% of the total grain weight, respectively, whereas that of wild-type was 74% (Table 2).

**Table 2:** Dehulled grain weight and starch content in mutant rice lines

Line	Grain weight^a^ per grain (mg)	Starch content^b^ per grain (mg)
*SS1/ss2a* ^*L*^ */SS3a* (Wild-type; Nipponbare)	20.3 ± 0.2^**^ (100)^c^	15.1 ± 1.1^**^ (74)^d^
*ss1* ^*L*^ */ss2a* ^*L*^ */SS3a* (*i2–1*)	20.2 ± 0.2^**^ (100)^c^	11.8 ± 0.5^**^ (58)^d^
*SS1/ss2a* ^*L*^ */ss3a* (*e1*)	18.8 ± 0.2^*,**^ (93)^c^	10.2 ± 0.8^*^ (54)^d^
*ss1* ^*L*^ */ss2a* ^*L*^ */ss3a* (*#6002*)	18.0 ± 0.2^*^ (89)^c^	9.9 ± 0.4^*^ (55)^d^

^a^Mean ± SE of 50 seeds

^b^Mean ± SE of three seeds

^c^Percentage of the wild-type

^d^Percentage of grain weight

^*^Significant differences between Nipponbare and mutant lines by the *t*-test at *P* < 0.05

^**^Significant differences between double mutant line and the other lines by the *t*-test at *P* < 0.05

### The effects of reduced SS activity on starch structure

Analyses of differences in the chain-length distribution patterns among the mutant lines and wild-type by performing capillary electrophoresis showed that the reduced SSI activity had an additive effect on the chain-length distribution of amylopectin in the *SS1/ss2a*^*L*^*/ss3a* background, with a slight synergistic enhancement [14, 15]. To further investigate the structure of starch in the double mutant lines, we subjected the debranched starch and purified amylopectin to gel filtration chromatography and calculated the apparent amylose content, extra-long chain content, and true amylose content, as well as the proportion of long chains and short chains of amylopectin (Table 3). Amylose plus extra-long chain, amylopectin long chains, and amylopectin short chains were eluted in Fractions I, II, and III, respectively, by chromatography (Additional file 5). The true amylose content was calculated by subtracting the extra-long chain content from the apparent amylose content (Table 3). Since amylose content is affected by temperature during seed development [35], we analyzed samples from two different years (2009 and 2013; Table 3).

**Table 3:** Composition of carbohydrate (weight %) in endosperm starch fractions separated by gel filtration chromatography

		Fr. I (%)^a^		Fr. II (%)		Fr. III (%)		III/II		Apparent amylose content (%)^e^	Apparent amylose content (mg/grain)^f^	True amylose content (%)^h^
Line		2009	2013	2009	2013	2009	2013	2009	2013			
*SS1/ss2a* ^*L*^ */SS3a* (wild-type; Nipponbare)	Starch^b^	20.3 ± 0.4^d^	18.8 ± 0.5	20.4 ± 0.2	22.5 ± 0.4	59.2 ± 0.5	58.7 ± 0.4	2.9 ± 0.1	2.6 ± 0.1	18.8	2.84 (100)^g^	17.1
	Amylopectin^c^	–	1.7 ± 0.3	–	21.5 ± 0.7	–	57.7 ± 1.0	–	2.7 ± 0.1			
*ss1* ^*L*^ */ss2a* ^*L*^ */SS3a* (*i2–1*)	Starch	No data	21.5 ± 0.6 ^*, **^	No data	21.2 ± 0.8 ^**^	No data	57.3 ± 0.5 ^**^	No data	2.7 ± 0.1 ^**^	21.5	2.54 (89) ^g^	19.9
	Amylopectin	–	1.6 ± 0.1 ^**^	–	20.9 ± 0.6 ^*, **^	–	57.2 ± 0.5 ^**^	–	2.7 ± 0.1 ^**^			
*SS1/ss2a* ^*L*^ */ss3a* (*e1*)	Starch	29.8 ± 0.2 ^*, **^	29.4 ± 0.6 ^*^	13.0 ± 0.2 ^*^	13.0 ± 0.3 ^*, **^	57.2 ± 0.4 ^*, **^	57.7 ± 0.3 ^**^	4.4 ± 0.1 ^*, **^	4.5 ± 0.1 ^*, **^	29.4	3.00 (106) ^g^	27.4
	Amylopectin	–	2.0 ± 0.1 ^*, **^	–	12.8 ± 0.5 ^*^	–	56.4 ± 0.7	–	4.4 ± 0.1 ^*, **^			
*ss1* ^*L*^ */ss2a* ^*L*^ */ss3a* (*#6002*)	Starch	34.0 ± 0.9 ^*^	30.3 ± 0.3 ^*^	13.8 ± 0.6 ^*^	14.8 ± 0.5 ^*^	52.2 ± 0.4 ^*^	54.9 ± 0.4 ^*^	3.8 ± 0.1 ^*^	3.7 ± 0.1 ^*^	30.3	3.00 (106) ^g^	27.1
	Amylopectin	–	3.2 ± 0.2 ^*^	–	14.2 ± 0.6 ^*^	–	54.5 ± 0.3 ^*^	–	3.8 ± 0.2 ^*^			

^a^Three fractions (Fr. I, II, and III) were divided at troughs of the carbohydrate content curve, as detected by refractive index detectors (Fig. 5)

^b^Total carbohydrate content = 100%

^c^Areas of Fr. II and Fr. III of amylopectin were superimposed onto those of the starch, and the amount of amylopectin (extra-long chain) in Fr. I was calculated

^d^Mean ± SE of three replicates

^e^Apparent amylose content (%) = Fr. I of starch in 2013

^f^Apparent amylose content (mg/grain) = (starch content (mg/grain) in Table 2) x (Apparent amylose content (%) in 2013, Table 3)

^g^Percentage of the wild-type

^h^True amylose content (%) = apparent amylose content (Fr. I of starch in 2013) – extra-long chains (Fr. I of amylopectin in 2013)

^*^Significant differences between Nipponbare and mutant lines by the *t*-test at *P* < 0.05

^**^Significant differences between double mutant line and the other lines by the *t*-test at *P* < 0.05

The apparent amylose content of *ss1*^*L*^*/ss2a*^*L*^*/SS3a* (21.5%) was slightly higher than that of wild-type (18.8%), whereas the apparent amylose content of *SS1/ss2a*^*L*^*/ss3a* was 1.6 times higher than that of wild-type (Table 3). These results are consistent with previous reports [13, 14, 36]. The apparent amylose content of *ss1*^*L*^*/ss2a*^*L*^*/ss3a* from 2013 was slightly higher than that of *SS1/ss2a*^*L*^*/ss3a*, but this different was not significant. The extra-long chain content of *ss1*^*L*^*/ss2a*^*L*^*/ss3a* was significantly higher than that of the other lines (Table 3).

SSIIIa elongates amylopectin long chains connecting clusters of amylopectin, as the levels of these chains with DP ≥30 are significantly reduced in *ss3a* [13]. The ratio of short to long chains of amylopectin (Fraction III/Fraction II) in endosperm starch was highest in *SS1/ss2a*^*L*^*/ss3a* among the lines tested in both years examined. This ratio was significantly higher in *ss1*^*L*^*/ss2a*^*L*^*/ss3a* than in wild-type and *ss1*^*L*^*/ss2a*^*L*^*/SS3a* but lower than that of *SS1/ss2a*^*L*^*/ss3a* (Table 3).

## Discussion

Reduction of three major SS isozymes responsible for amylopectin synthesis in rice endosperm increased the levels of AGPase and GBSSI, which raised the amylose content.

SSI, SSIIa and SSIIIa are the major SS isozymes responsible for amylopectin biosynthesis in rice endosperm and are also commonly found in cereal grains [3]. The rice mutant lacks both SSI and SSIIIa and possesses low SSIIa activity (*ss1/ss2a*^*L*^*/ss3a*) and was previously shown to be sterile, while rice plants with either SSI or SSIIIa generated heterozygous seeds [14]. In this study, the rice mutant line (*ss1*^*L*^*/ss2a*^*L*^*/ss3a*), which has low SSI and SSIIa activities and lacks SSIIIa, was analyzed for its starch properties, pleiotropic effects of other starch biosynthetic enzymes, and their interactions.

*ss1*^*L*^*/ss2a*^*L*^*/ss3a* yielded opaque mature seeds (Fig. 6) and maintained 89% of the seed weight of wild-type (Table 2). Although the amount of starch was slightly decreased, the apparent amylose content within starch increased to 30–34% compared with that of wild-type (ca. 20%) and the parental lines (*ss1*^*L*^*/ss2a*^*L*^*/SS3a* was 22% and *SS1/ss2a*^*L*^*/ss3a* was ca. 30%; Table 3). Weight of apparent amylose content per grain was calculated by (starch content; Table 2) x (apparent amylose content %; Table 3) and that 2.84 mg in wild-type, 2.54 mg in *ss1*^*L*^*/ss2a*^*L*^*/SS3a*, and 3.00 mg (106% of wild-type) in *SS1/ss2a*^*L*^*/ss3a* and *ss1*^*L*^*/ss2a*^*L*^*/ss3a* (Table 3). These suggest that both amylose and ELC biosynthesis were enhanced in *SS1/ss2a*^*L*^*/ss3a* and *ss1*^*L*^*/ss2a*^*L*^*/ss3a*.

An increase in amylose content is often considered the outcome of reduced amylopectin synthesis, which leads to an increase in the substrate providing AGPase and favors amylose synthesis by GBSSI [13, 14, 31, 36]. The AGPase activity in a maize SSIII-deficient mutant (*du1*) is higher than that of wild-type [37]. In rice, SSI, SSIIIa, and BEIIb deficiencies lead to increased AGPase activity [13, 14, 28, 36]. The present study also showed increased AGPase and GBSSI amounts (Fig. 2) and AGPase activity (Table 1) in *SS1/ss2a*^*L*^*/ss3a*, consistent with a previous study [14], and in *ss1*^*L*^*/ss2a*^*L*^*/ss3a*. The increase in the amount of AGPase and GBSSI protein (Fig. 2) suggests that these enzymes are regulated at the transcriptional level.

GBSSI is responsible for amylose biosynthesis, and typical japonica rice cultivars contain a SNP at the boundary between exon 1 and intron 1 of the *GBSSI* gene compared with the indica rice cultivars. This SNP results in incorrect splicing of intron 1 and produces incomplete *GBSSI* mRNAs [38]. The mRNA processing efficiency of GBSSI has been demonstrated to respond differently to the temperature during seed development [39]. It is known that the amounts of mature *GBSSI* mRNA decrease at high temperatures (32 °C) compared with the normal (25 °C) temperature during seed development, resulting in a low amylose content in the endosperm starch compared with that grown at a low temperature (18 °C) [40]. The average temperature at 30 days after flowering in the study area, Akita, was 18.8 °C and 21.4 °C in 2009 and 2013, respectively [41]. The apparent amylose content of every rice line harvested in 2009 was higher than that in 2013 (Table 3). In particular, the apparent amylose content of *ss1*^*L*^*/ss2a*^*L*^*/ss3a* (34.0%) was significantly higher than that of *SS1/ss2a*^*L*^*/ss3a* (29.8%; Table 3 in 2009). The significantly higher AGPase activity resulting from the reduction of SSI activity and SSIIIa deficiency (Table 1) should lead to a high concentration of ADP-glucose in the amyloplast, although we did not estimate the level of ADP-glucose. GBSSI has a higher Km for ADP-glucose than the other soluble SS isozymes in potato [42]. If this is the same in the case of rice, amylose synthesis would also be enhanced in rice endosperm. We suspect that the high GBSSI expression in *ss1*^*L*^*/ss2a*^*L*^*/ss3a* amyloplasts under high ADP-glucose concentrations leads to efficient amylose biosynthesis, which occurs when temperatures are not high during seed development.

### Reduction of major SS isozymes in rice endosperm and the pleiotropic effects of other starch biosynthetic enzymes and their interactions

Starch biosynthetic enzymes in cereal are known to interact with each other and to form protein-protein complexes [16, 18, 19, 21, 23, 43]. SSI forms protein complexes with SSIIa and BEI or BEIIb in developing seeds of rice, wheat, maize and barley [16, 17, 20–23]. The mutual and synergistic interaction of SSI with BE isozymes has also been shown via in vitro studies of recombinant enzymes in rice [34] and *Arabidopsis* [44]. SSIIIa forms high-molecular-weight protein complexes (> 700 kDa) and interacts with SSIIa, BEI and BEIIb in the developing endosperm of wild-type rice [23]. SSIII (corresponding to rice SSIIIa) in maize developing kernels is also known to form protein complexes containing plastidial AGPase, pyruvate orthophosphate dikinase, SSIIa, and BE II isozymes [19].

The present study compared the elution patterns (Fig. 3 and Additional file 1) and activities (Fig. 4 and Additional file 2) of starch biosynthetic proteins separated by gel filtration chromatography using soluble extract from the developing rice seeds of *ss1*^*L*^*/ss2a*^*L*^*/ss3a*, *ss1*^*L*^*/ss2a*^*L*^*/SS3a*, *SS1/ss2a*^*L*^*/ss3a*, and wild-type (*SS1/ss2a*^*L*^*/SS3a*). The amounts and activity of SSI in the eluted fractions from gel filtration column were generally correlated (Figs. 3 and 4); the highest level of SSI was found in *SS1/ss2a*^*L*^*/ss3a*, followed by wild-type, *ss1*^*L*^*/ss2a*^*L*^*/ss3a*, and *ss1*^*L*^*/ss2a*^*L*^*/SS3a* was the lowest. More specifically, greater amounts and activities of SSI were found at a high molecular weight (> 300 kDa: Fr. 2–8) in *SS1/ss2a*^*L*^*/ss3a* compared with the other lines (Figs. 3 and 4). This finding suggests that SSI may be incorporated into the high-molecular-weight protein complexes (> 440 kDa), in which SSIIIa was eluted in wild-type, possibly to compensate for the absence of SSIIIa. Considering that the expression levels and activities of SSI were significantly lower in *ss1*^*L*^*/ss2a*^*L*^*/SS3a* and *ss1*^*L*^*/ss2a*^*L*^*/ss3a* compared with wild-type (Figs. 1 and 2), the ratio of SSI eluted in Fr. 8–12 was higher than that in Fr.4–7 in *ss1*^*L*^*/ss2a*^*L*^*/SS3a* and *ss1*^*L*^*/ss2a*^*L*^*/ss3a* (Fig. 3). Since maize SSI is known to form an approximately 230 kDa trimeric SSI-SSIIa-BE protein complex [21], it is possible that SSI may be enriched in Fr. 8–12 in *ss1*^*L*^*/ss2a*^*L*^*/SS3a* and *ss1*^*L*^*/ss2a*^*L*^*/ss3a* to form a similar type of protein complex.

Unlike in *ss1*^*L*^*/ss2a*^*L*^*/SS3a*, *SS1/ss2a*^*L*^*/ss3a*, and wild-type, SSIIa was enriched in Fr. 8–10 but depleted in Fr.2–6 of *ss1*^*L*^*/ss2a*^*L*^*/ss3a* (Fig. 3). In the absence of SSIIIa and reduction of SSI in *ss1*^*L*^*/ss2a*^*L*^*/ss3a*, SSIIa may not be able to form high-molecular-weight complexes, which would elute in Fr. 1–7. Although it is yet to be investigated, it is possible that the SSIIa eluted in Fr. 8–10 of *ss1*^*L*^*/ss2a*^*L*^*/ss3a* may present alternative protein complexes such as the SSIIa-SSIIa-BE trimeric complex.

The SSIIa used in the present study was of japonica rice origin, which has four SNPs and shows less than 10% of the activity compared to indica type SSIIa [11].These results suggest that the presence rather than the activity of SSIIa may be important for the formation of protein-protein complexes.

Co-immunoprecipitation experiments showed that the association of BEI-BEIIb, BEIIa-Pho, BEI-PUL were observed in all the analyzed rice lines (Additional file 4); however, the interaction of SSI and SSIIa with other starch biosynthetic enzymes was reduced in *ss1*^*L*^*/ss2a*^*L*^*/SS3a*, *SS1/ss2a*^*L*^*/ss3a*, and *ss1*^*L*^*/ss2a*^*L*^*/ss3a* compared with wild–type (Fig. 5). Since gel filtration chromatography suggested the assembly of protein complexes for the remaining SS isozymes in all analyzed rice lines, there is some possibility that alternatively assembled protein complexes with SS isozymes in *ss1*^*L*^*/ss2a*^*L*^*/SS3a*, *SS1/ss2a*^*L*^*/ss3a*, and *ss1*^*L*^*/ss2a*^*L*^*/ss3a* may be more susceptible in the current experimental conditions.

Concurrently, the location and type of amino acid substitution in the specific starch biosynthetic enzymes likely have different effect on protein complex formation, depending on the protein structure of the counterpart enzymes.

Altered branch structures of amylopectin in *ss1*^*L*^*/ss2a*^*L*^*/ss3a*, *ss1*^*L*^*/ss2a*^*L*^*/SS3a*, and *SS1/ss2a*^*L*^*/ss3a* shown by capillary electrophoresis suggest [13] that alternatively formed protein complexes may not perfectly mimic the choice of glucan primers and the final length of amylopectin branches; the latter may be more dependent on the three-dimensional structure of individual SS isozymes. Future analyses of the reconstitution of protein complexes using purified recombinant enzymes are necessary.

### Speculation regarding the alternatively formed protein–protein complexes in mutant rice endosperms

**Fig. 7:**
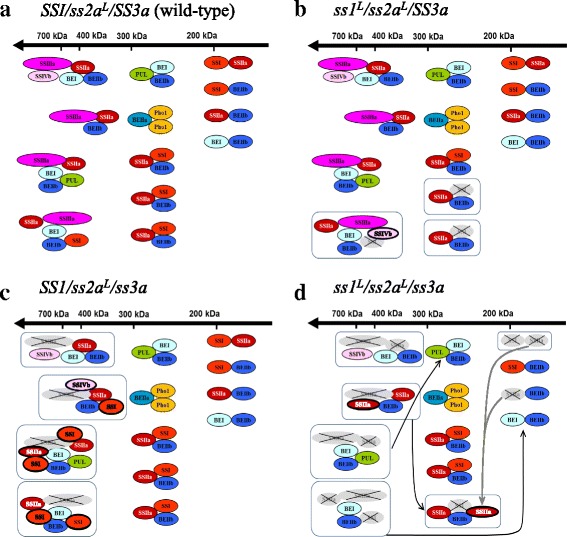
Schematics of speculated starch biosynthetic protein complexes in wild-type (*SS1/ss2a*^*L*^*/SS3a*), *ss1*^*L*^*/ss2a*^*L*^*/SS3a*, *SS1/ss2a*^*L*^*/ss3a* and *ss1*^*L*^*/ss2a*^*L*^*/ss3a*. Selected combinations of predicted protein-protein complexes in developing seed of japonica rice (*SS1/ss2a*^*L*^*/SS3a*) [23] are shown as wild-type (**a**). Alterations of protein complexes in *ss1*^*L*^*/ss2a*^*L*^*/SS3a* (**b**), *SS1/ss2a*^*L*^*/ss3a* (**c**) and *ss1*^*L*^*/ss2a*^*L*^*/ss3a* (**d**) are illustrated. Boxes indicate an altered protein complex. SS isozymes that were missing or reduced compared with wild-type are indicated in gray. Newly recruited speculated SS isozymes are indicated with a bold oval. Black arrows indicate a reduced molecular weight due to missing enzymes. Gray arrows indicate recruitment of enzymes. Note that all lines used in this study have low SSIIa activity

Possible combinations of starch biosynthetic protein complexes in *SS1/ss2a*^*L*^*/SS3a*, *ss1*^*L*^*/ss2a*^*L*^*/SS3a*, *SS1/ss2a*^*L*^*/ss3a*, and *ss1*^*L*^*/ss2a*^*L*^*/ss3a* were speculated (Fig. 7) using the results of gel filtration chromatography (Fig. 3, Additional file 1, and [23]), although some of the interactions were yet to be confirmed in the current immuno-precipitation condition (Fig. 5, Additional file 4, and [23]). The selected predicted protein–protein complexes found in developing seed of wild-type japonica rice (*SS1/ss2a*^*L*^*/SS3a*) [23] are shown in Fig. 7a. Multiple types of protein complexes with different sizes are predicted to form in the wild-type, such as low-molecular-weight protein complexes (approximately 200–300 kDa) containing SSI-SSIIa-BEIIb (Fig. 7) [23].

Alteration of the protein complexes in *ss1*^*L*^*/ss2a*^*L*^*/SS3a* is outlined in Fig. 7b. When the expression of SSI was reduced, the amounts of SSI in both high and low-molecular-weight fractions reduced, whereas the amounts of SSIVb > 700 kDa increased (Fig. 3). It is speculated that the reduction of SSI in high-molecular-weight complexes could potentially be complemented by SSIVb in *ss1*^*L*^*/ss2a*^*L*^*/SS3a* to form alternative protein complexes (Fig. 7b). The amounts of SSI-SSIIa-BEIIb trimeric complex could also be reduced in *ss1*^*L*^*/ss2a*^*L*^*/SS3a* (Fig. 7b).

Loss of SSIIIa led to increased expression of SSI in *SS1/ss2a*^*L*^*/ss3a*. The amount of SSI > 440 kDa noticeably increased in *SS1/ss2a*^*L*^*/ss3a* compared to that in wild-type (Fig. 3). The elution of SSIIa and SSIVb > 700 kDa was also increased in *SS1/ss2a*^*L*^*/ss3a* (Fig. 3). This suggests that alternative high-molecular-weight protein complexes enriched with SSI, SSIIa, and/or SSIVb were assembled in *SS1/ss2a*^*L*^*/ss3a* (Fig. 7c).

Reduction of SSI and loss of SSIIIa in *ss1*^*L*^*/ss2a*^*L*^*/ss3a* led to a considerable decrease in the amounts of eluted SSI and SSIIa > 440 kDa (Fig. 3). This suggests that high-molecular-weight protein complexes were not assembled in *ss1*^*L*^*/ss2a*^*L*^*/ss3a*, and alternatively, protein complexes with a lower molecular weight, such as the PUL-BEI-BEIIb and/or SSIIa-SSIIa-BEIIb complex, might be formed in *ss1*^*L*^*/ss2a*^*L*^*/ss3a* (Fig. 7d).

Taken together, when either SSI and/or SSIIIa were absent in the conditions of low SSIIa activity in developing rice seed, the composition of starch biosynthetic enzyme complexes involved in amylopectin biosynthesis were complemented in SS isozymes to form altered protein complexes. Hence, *ss1*^*L*^*/ss2a*^*L*^*/ss3a* was able to minimize the reduction of carbon storage and avoided sterility, although the branch length of amylopectin was different from that of the wild-type.

## Conclusions

Although the simultaneous reduction of three major SSs, SSI, SSIIa and SSIIIa, resulted in less amylopectin in *ss1*^*L*^*/ss2a*^*L*^*/ss3a* endosperm, a significant amount of amylopectin was synthesized and amylose content was increased in *ss1*^*L*^*/ss2a*^*L*^*/ss3a*. Protein complex formation of starch biosynthetic enzymes was altered in terms of the size of the protein complexes containing SSI, SSIIa, and SSIVb, as determined by gel filtration chromatography. In addition, co-immuno-precipitation analyses showed that the associations of SS isozymes with other starch biosynthetic enzymes were weaker or reduced in the current experimental conditions.

The present study indicated the importance of formation of starch biosynthetic protein complexes in developing rice endosperms, even when there is a deficiency of essential SS isozymes, by exchanging and complementing the composition of protein complexes within the same enzyme family (SS isozyme) to maximize the storage of photosynthetic products as starch. The substitution of starch biosynthetic protein complexes in BE isozymes seen in maize developing kernels that lack BEIIb could also be a similar phenomenon [20, 24]. Therefore, the present study enhances our understanding of the control of the amount and structure of starch in relation to the formation of starch biosynthetic protein complexes..

## Additional file

Additional file 1: Molecular weight distributions of BE isozymes from developing rice endosperm determined by gel filtration chromatography. Soluble proteins were separated by gel filtration chromatography. Fractions were denatured and separated by SDS-PAGE, and immuno-blotting was performed using the indicated antibodies. (PDF 903 kb)

Additional file 2: SS activities were visualized by non-denaturing zymograms using rice starch biosynthetic enzymes separated by gel filtration chromatography from SS1/ss2aL/ss3a. Gel was incubated in the presence of 1 mM ADP-glucose (A) and in the absence of ADP-glucose (B). Black arrowhead shows the SSI activity. Gray arrowheads represent glycosyl hydrolase or glucan transferase activities. White arrowheads are SS activity bands found only in ss3a. (PDF 903 kb)

Additional file 3: Immuno-blotting of SS isozymes resolved by non-denaturing PAGE using the fractions obtained from gel filtration chromatography. Antibodies used for immuno-blotting are indicated. Black arrowheads indicate the same migration distances as the main stained activity bands shown in Fig. 4, and white arrowheads correspond to the activity bands found in the zymogram of SS1/ss2aL/ss3a in Fig. 4. (PDF 902 kb)

Additional file 4: Analyses of protein-protein interactions between rice starch biosynthetic isozymes by co-immunoprecipitation. Immuno-precipitation experiments were performed using the isozyme specific antibodies indicated above and the soluble protein extract from SS1/ss2aL/SS3a (WT), ss1L/ss2aL/SS3a, SS1/ss2aL/ss3a and ss1L/ss2aL/ss3a. Immuno-blotting was performed using the antibodies indicated on the right. (PDF 903 kb)

Additional file 5: Size separation of debranched endosperm starch and purified amylopectin by gel filtration showing the amylose content in fraction I.Gel filtration chromatography was performed for debranched endosperm starch and purified amylopectin using the SS1/ss2aL/SS3a (Nipponbare; A), ss1L/ss2aL/SS3a (B), SS1/ss2aL/ss3a (C), and ss1L/ss2aL/ss3a (D) harvested in 2013. Each graph shows typical elution profiles of isoamylase-debranched starch (blue lines) and purified amylopectin (red lines). Each fraction (Fr. I, II, and III) is separated according to the carbohydrate content curve determined by refractive index detectors (left Y-axis). The panels show one typical data set (of at least three replicates prepared from starch and purified amylopectin). (PDF 901 kb)

## Abbreviations

ADP-glucose: Adenosine diphosphate-glucose
AGPase: ADP-glucose pyrophosphorylase
BE: Starch branching enzyme
DAF: Days after flowering
DBE: Debranching enzymes
DP: Degree of polymerization
Fr: Fraction
GBSSI: Granule-bound starch synthase I
ISA: Isoamylase
PAGE: Polyacrylamide gel electrophoresis
Pho: Phosphorylase
PUL: Pullulanase
SDS: Sodium dodecyl sulfate
SNPs: Single nucleotide polymorphisms
SS: Starch synthase
