# Supplementary figures and images for: Analyses of starch biosynthetic protein complexes and starch properties from developing mutant rice seeds with minimal starch synthase activities
Source: BMC Plant Biol. 2018 Apr 10;18:59. doi: 10.1186/s12870-018-1270-0 (PMC5894220; doi:10.1186/s12870-018-1270-0)

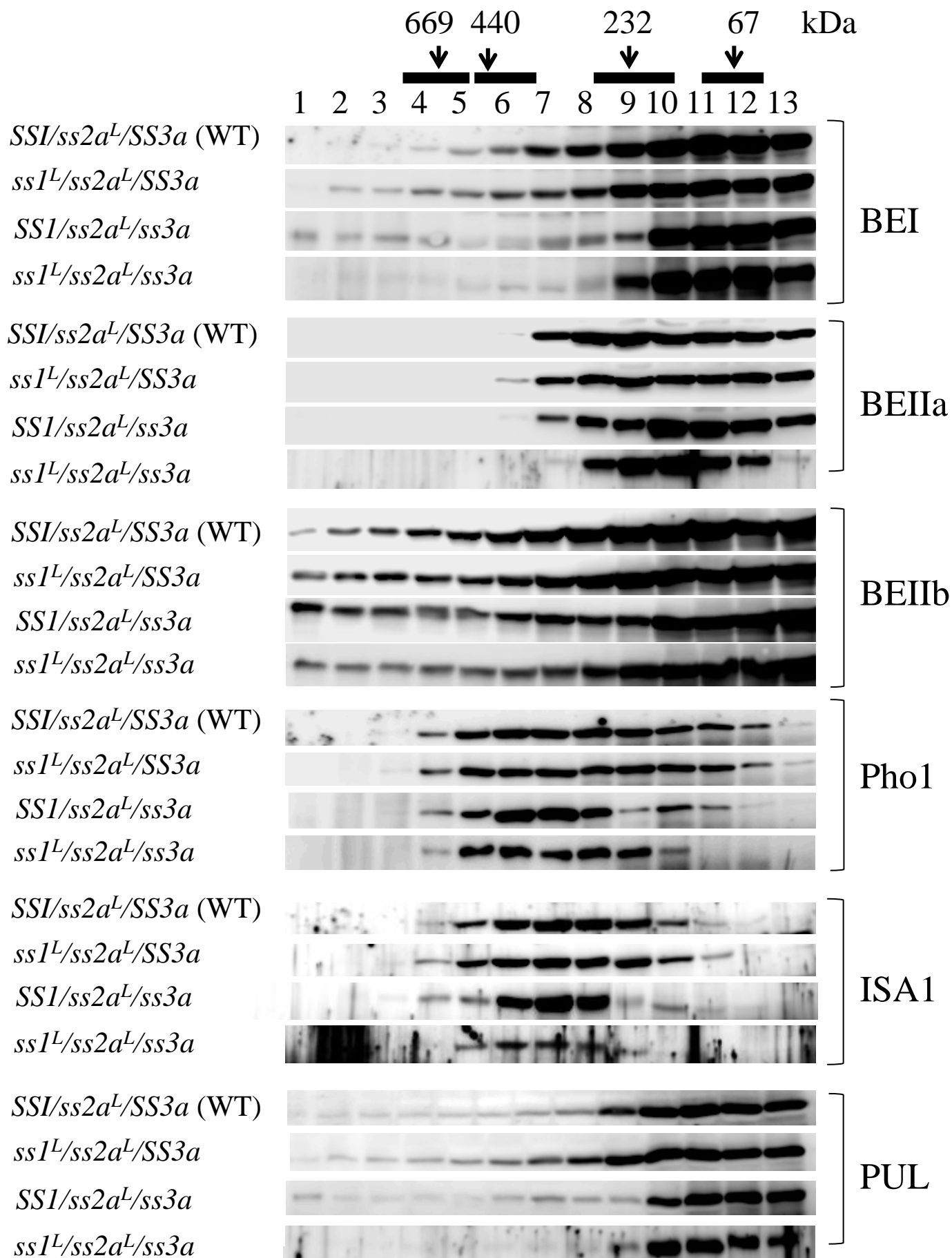

Supplement: Supplementary file 1 — Molecular weight distributions of BE isozymes from developing rice endosperm determined by gel filtration chromatography. Soluble proteins were separated by gel filtration chromatography. Fractions were denatured and separated by SDS-PAGE, and immuno-blotting was performed using the indicated antibodies. (PDF 903 kb) [file 12870_2018_1270_MOESM1_ESM.pdf]

(A) 1mM ADP-glucose

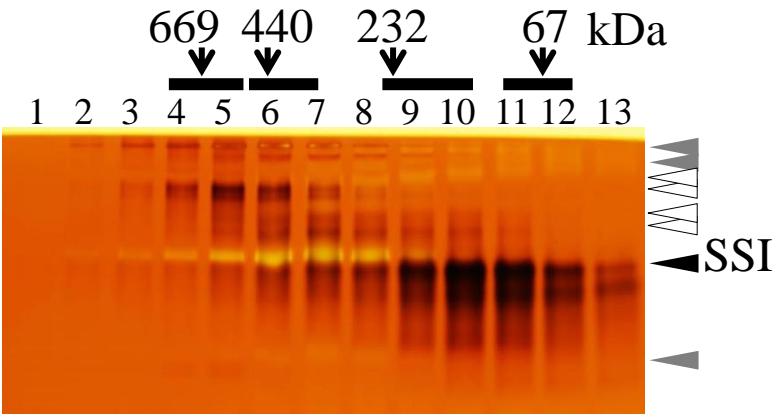

(B) 0 mM ADP-glucose

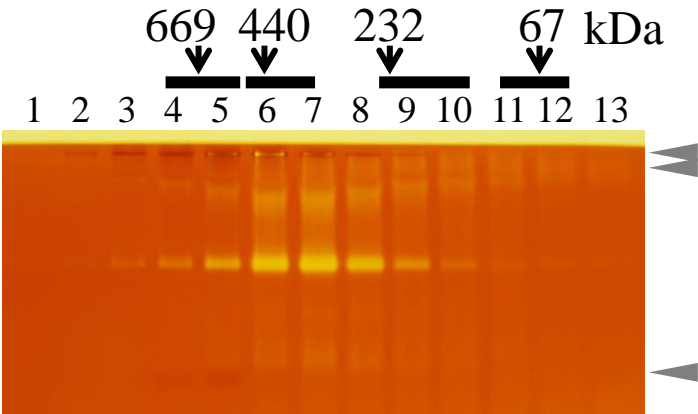

Supplement: Supplementary file 2 — SS activities were visualized by non-denaturing zymograms using rice starch biosynthetic enzymes separated by gel filtration chromatography from SS1/ss2aL/ss3a. Gel was incubated in the presence of 1 mM ADP-glucose (A) and in the absence of ADP-glucose (B). Black arrowhead shows the SSI activity. Gray arrowheads represent glycosyl hydrolase or glucan transferase activities. White arrowheads are SS activity bands found only in ss3a. (PDF 903 kb) [file 12870_2018_1270_MOESM2_ESM.pdf]

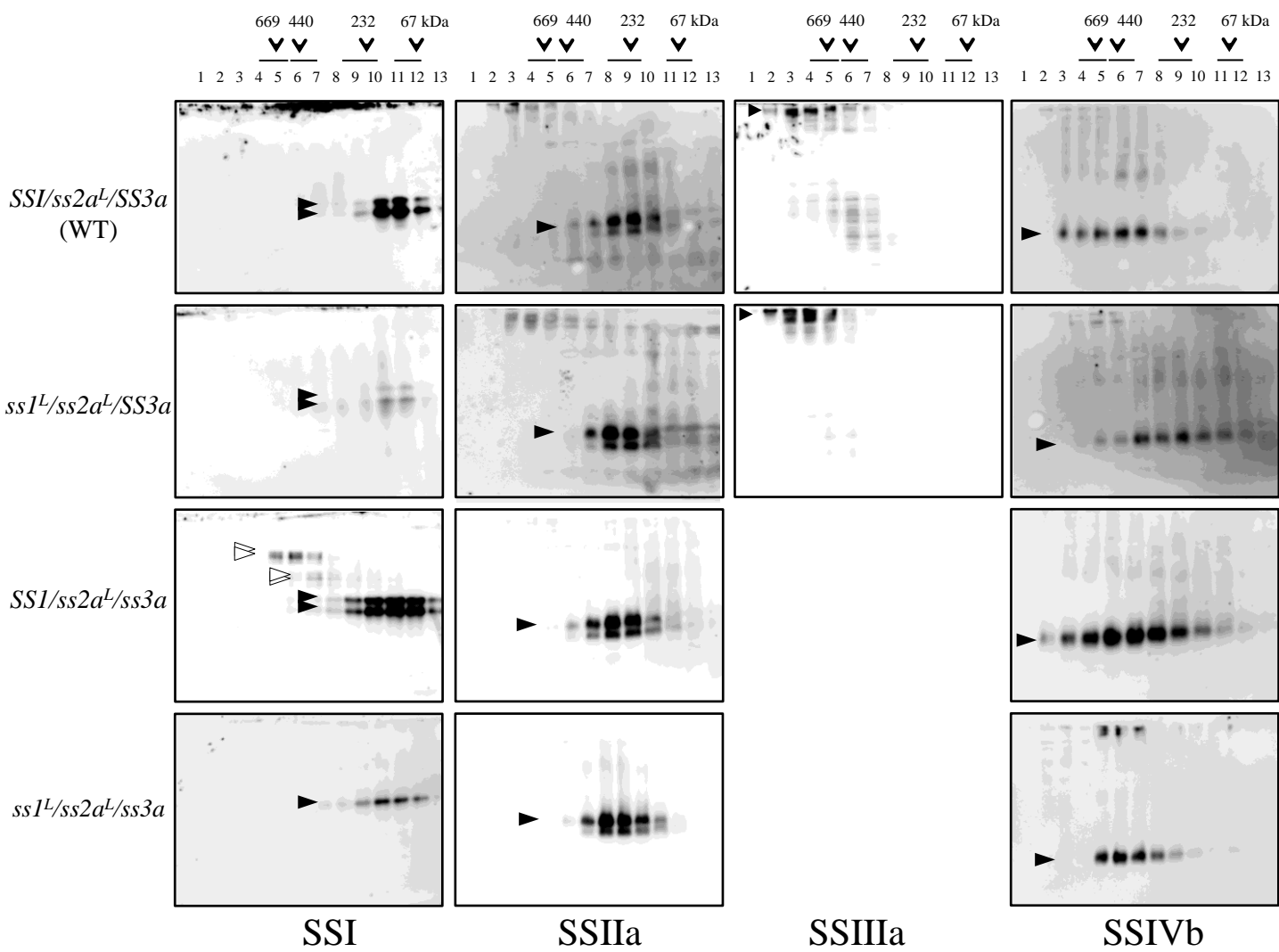

Supplement: Supplementary file 3 — Immuno-blotting of SS isozymes resolved by non-denaturing PAGE using the fractions obtained from gel filtration chromatography. Antibodies used for immuno-blotting are indicated. Black arrowheads indicate the same migration distances as the main stained activity bands shown in Fig. 4, and white arrowheads correspond to the activity bands found in the zymogram of SS1/ss2aL/ss3a in Fig. 4. (PDF 902 kb) [file 12870_2018_1270_MOESM3_ESM.pdf]

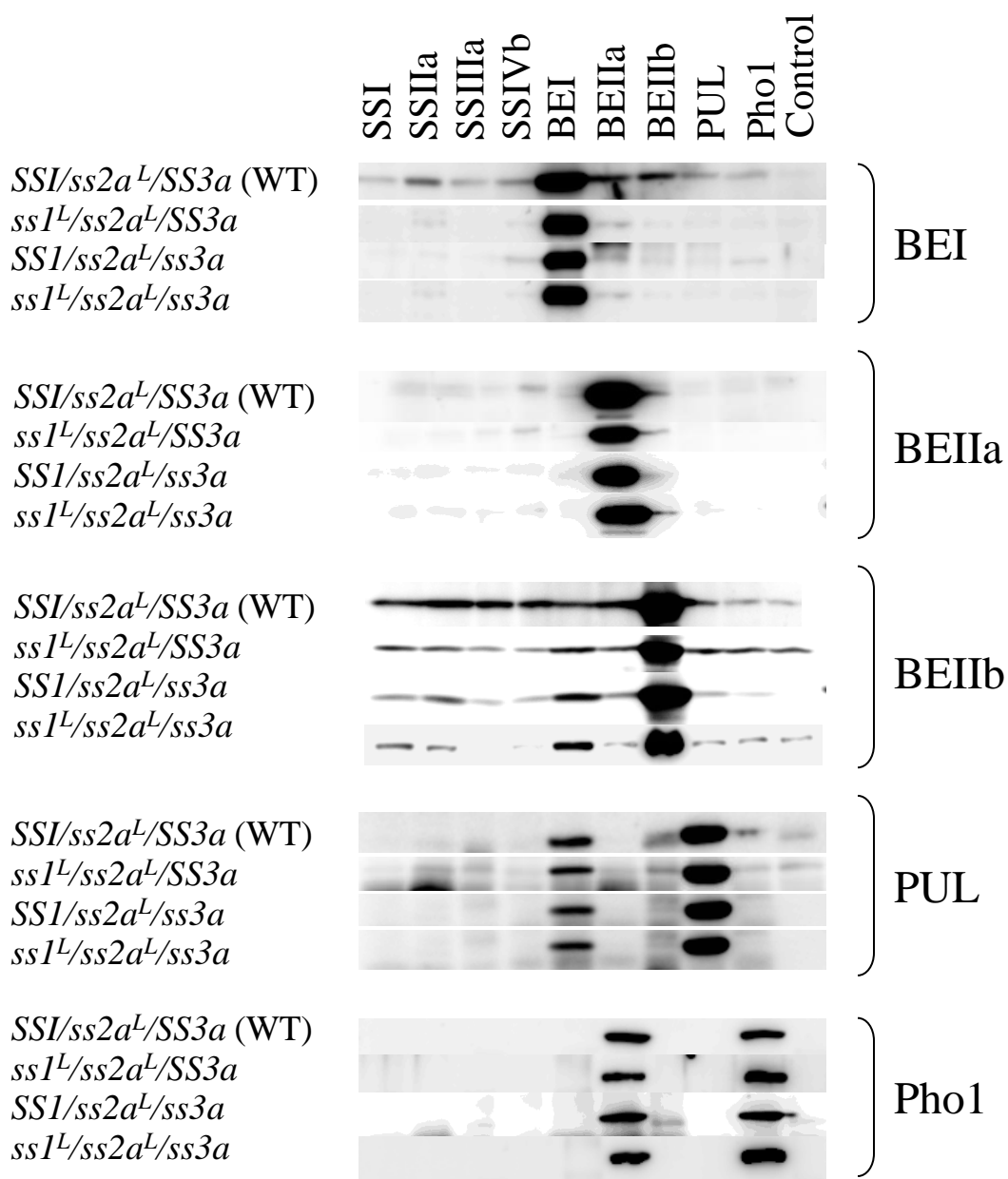

Supplement: Supplementary file 4 — Analyses of protein-protein interactions between rice starch biosynthetic isozymes by co-immunoprecipitation. Immuno-precipitation experiments were performed using the isozyme specific antibodies indicated above and the soluble protein extract from SS1/ss2aL/SS3a (WT), ss1L/ss2aL/SS3a, SS1/ss2aL/ss3a and ss1L/ss2aL/ss3a. Immuno-blotting was performed using the antibodies indicated on the right. (PDF 903 kb) [file 12870_2018_1270_MOESM4_ESM.pdf]

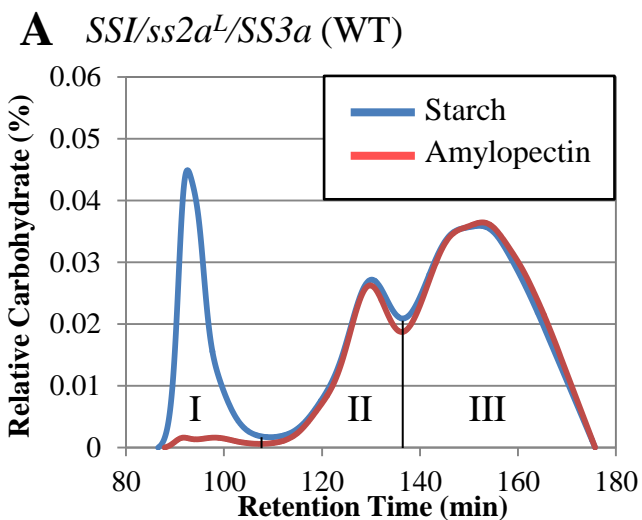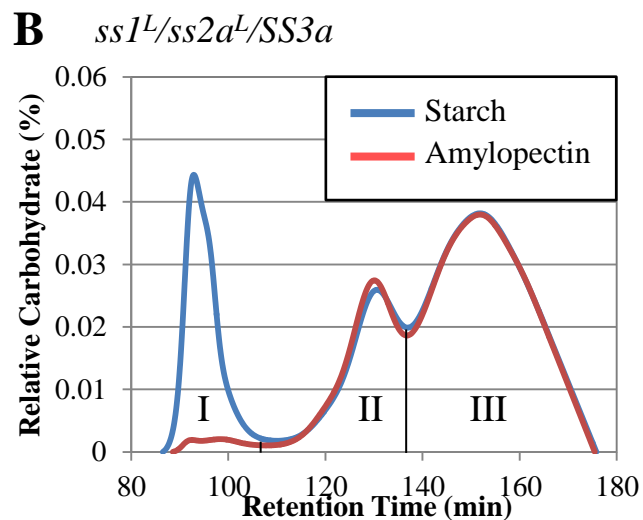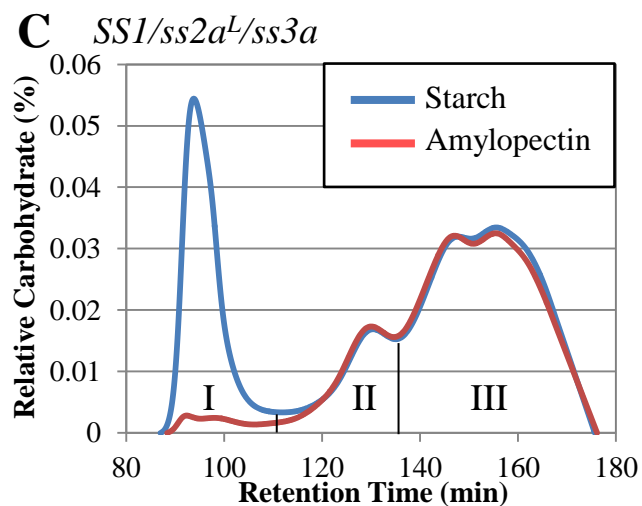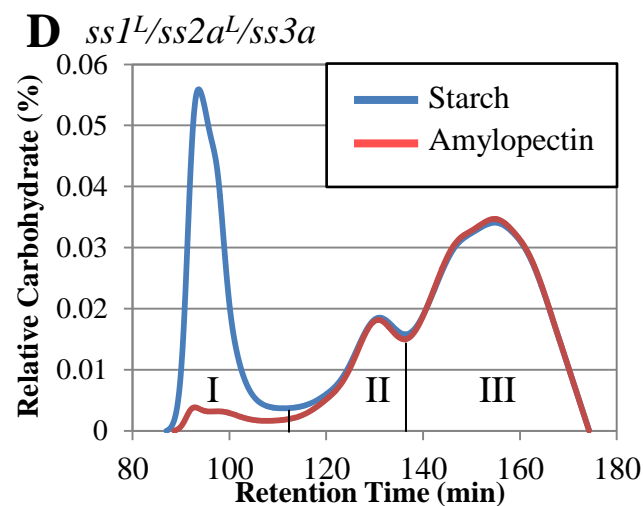

Supplement: Supplementary file 5 — Size separation of debranched endosperm starch and purified amylopectin by gel filtration showing the amylose content in fraction I.Gel filtration chromatography was performed for debranched endosperm starch and purified amylopectin using the SS1/ss2aL/SS3a (Nipponbare; A), ss1L/ss2aL/SS3a (B), SS1/ss2aL/ss3a (C), and ss1L/ss2aL/ss3a (D) harvested in 2013. Each graph shows typical elution profiles of isoamylase-debranched starch (blue lines) and purified amylopectin (red lines). Each fraction (Fr. I, II, and III) is separated according to the carbohydrate content curve determined by refractive index detectors (left Y-axis). The panels show one typical data set (of at least three replicates prepared from starch and purified amylopectin). (PDF 901 kb) [file 12870_2018_1270_MOESM5_ESM.pdf]
